# Impaired Fas-Fas Ligand Interactions Result in Greater Recurrent Herpetic Stromal Keratitis in Mice

**DOI:** 10.1155/2015/435140

**Published:** 2015-10-04

**Authors:** Xiao-Tang Yin, Tammie L. Keadle, Jessicah Hard, John Herndon, Chloe A. Potter, Chelsea R. Del Rosso, Thomas A. Ferguson, Patrick M. Stuart

**Affiliations:** ^1^Department of Ophthalmology, Saint Louis University School of Medicine, St. Louis, MO 63104, USA; ^2^Department of Ophthalmology & Visual Sciences, Washington University School of Medicine, St. Louis, MO 63110, USA

## Abstract

Herpes simplex virus-1 (HSV-1) infection of the cornea leads to a potentially blinding condition termed herpetic stromal keratitis (HSK). Clinical studies have indicated that disease is primarily associated with recurrent HSK following reactivation of a latent viral infection of the trigeminal ganglia. One of the key factors that limit inflammation of the cornea is the expression of Fas ligand (FasL). We demonstrate that infection of the cornea with HSV-1 results in increased functional expression of FasL and that mice expressing mutations in Fas (*lpr*) and FasL (*gld*) display increased recurrent HSK following reactivation compared to wild-type mice. Furthermore, both *gld* and *lpr* mice took longer to clear their corneas of infectious virus and the reactivation rate for these strains was significantly greater than that seen with wild-type mice. Collectively, these findings indicate that the interaction of Fas with FasL in the cornea restricts the development of recurrent HSK.

## 1. Introduction

Herpetic stromal keratitis (HSK) is a potentially blinding corneal inflammation that accompanies herpes simplex virus (HSV) infection of the eye. The disease course in HSK begins with a primary infection by HSV followed by a period during which the virus enters latency in sensory and autonomic ganglia. Many studies have shown that clinical disease is the result of a cocktail of inflammatory cells, consisting of PMNs, macrophages, and T cells (both CD4^+^ and CD8^+^) that are recruited to the corneas of patients with HSK [1–4].

In the face of this potentially blinding inflammatory attack, the cornea has the ability to reduce inflammation. This includes the presence of immunosuppressive factors such as TGF-*β* [5], lack of vascularization [6, 7], and the presence of Fas ligand (FasL) [8–14].

Studies from our laboratory as well as the laboratories of others have demonstrated that the presence of FasL in the eye is an important barrier to both inflammatory cells [8, 9, 12] and new blood vessels [10, 11, 13, 14]. In fact, we know that control of inflammation is required for the immune privilege of the eye [8, 9]. FasL expressed on ocular tissues induces apoptosis in Fas^+^ lymphoid cells that invade the eye in response to viral infection [8] or corneal grafting [11, 12, 14]. FasL expressed in the retina and the cornea also controls new vessel growth beneath the retina and in the cornea by inducing apoptosis of Fas-expressing vascular endothelial cells [15–17]. These studies clearly indicate that the presence of FasL in ocular tissues restricts inflammatory responses.

Recently we published that the interaction of Fas with FasL is an important factor in controlling HSK during acute infection of the cornea [18]. We demonstrated that mice expressing mutations in Fas (*lpr*) or FasL (*gld*) experience significantly worse ocular disease than do wild-type mice regardless of mouse or viral strain [18]. However, since acute infection rarely leads to clinical disease in humans and factors important in acute infection do not display an analogous role during recurrent infection [19], we thought it is very important to address the role that Fas and FasL play during recurrent disease when the virus is reactivated from latency. In order to address the role of Fas-FasL interactions during recurrent HSK, we have evaluated this interaction in a mouse model of induced recurrent HSK. We report here that mice that are defective in either Fas or FasL experience increased recurrent HSK disease following infection with HSV-1.

## 2. Materials and Methods

### 2.1. Virus and Cells

The virus used in these studies was the McKrae strain of HSV-1. A plaque-purified stock was grown and assayed on Vero cells in minimum essential medium with Earle's balanced salts (MEM-EBS) containing 5% fetal bovine serum, 100 U/mL penicillin, and 100 *μ*g/mL streptomycin [20]. Virus titers in eye swabs were determined by standard plaque assay [20].

### 2.2. Mice

Investigations with mice conformed to the Association for Research in Vision and Ophthalmology (ARVO) Statement for the Use of Animals in Ophthalmic and Vision Research. C57BL/6 (B6) and BALB/c mice were purchased from NCI. The B6Smn.C3-*Tnfsf6* ^*gld*^/J and B6.MRL-*Tnfsf6* ^*lpr*^/J mice were purchased from Jackson Labs and maintained in our colony. For the purposes of this paper we will refer to these mice as B6-*gld* and B6-*lpr*, respectively. We also bred the B6-*gld* and B6-*lpr* mice to BALB/c mice for a minimum of 12 generations. The resultant strains designation will be C.B6-*Tnfsf6* ^*gld*^ and C.B6-*Tnfsf6* ^*lpr*^ [21, 22]. However, we will refer to them as BALB-*gld* and BALB-*lpr*, respectively. In order to assure that these mice retain their mutations, tail DNA is isolated from individual mice and PCR tested for either the* gld* or the* lpr* mutation.

### 2.3. Infection of Mice

6–12-week-old mice were infected on the scarified cornea with 10^6^ PFU HSV-1 McKrae strain as previously described [23]. Each mouse received an intraperitoneal (IP) injection of 0.5 mL pooled human serum (Sigma Chemicals, St. Louis MO; ED50 for virus neutralization = 1 : 1600) concurrent with infection. Administration of pooled human serum which is the source of anti-HSV antibodies at the time of ocular infection has been shown to protect mice from death and corneal disease during primary infection, while allowing for the establishment of latency and subsequent reactivation of virus after corneal UV-B exposure. These human antibodies are undetectable at the time of UV-B irradiation 5 weeks after primary infection. HSV positive eye swabs obtained three days after application of virus confirm primary infection.

### 2.4. UV-B Irradiation and Virus Reactivation

Mice were reactivated from latency as previously described [24]. Briefly, the eyes of all latently infected mice were examined for corneal opacity before irradiation, and only animals with clear corneas were used. At least 5 weeks after primary infection, at which time human antibodies cannot be detected, the eyes of latently infected and control mock-infected mice were exposed to 250 mJ/cm^2^ of UV-B light using a TM20 Chromato-Vue transilluminator (UVP, Inc., San Gabriel, CA), which emits UV-B at a peak wavelength of 302 nm. Irradiated mice were swabbed with sterile cotton applicators from day 0 to day 7, unless otherwise indicated. The swab material was cultured on Vero cells, as described above, in order to detect recurrent virus shedding from the cornea. Reactivation was defined as the finding of any HSV positive eye swab on any days after UV-B exposure, with day 0 swabs serving as a control.

### 2.5. Clinical Evaluation

On the designated days after viral infection or UV-B reactivation, a masked observer examined mouse eyes through a binocular-dissecting microscope in order to score clinical disease. Stromal opacification was rated on a scale of 0 to 4, where 0 indicates clear stroma, 1 indicates mild stromal opacification, 2 indicates moderate opacity with discernible iris features, 3 indicates dense opacity with loss of defined iris detail except pupil margins, and 4 indicates total opacity with no posterior view. Corneal neovascularization was evaluated as described [20, 21] using a scale of 0–8, where each of four quadrants of the eye is evaluated for the amount of vessels that have grown into them. Periocular disease was measured in a masked fashion on a semiquantitative scale as previously described [25].

### 2.6. Viral Tittering from Tissues

Eye swab material was collected and assayed for virus by standard plaque assay as previously described [20]. Trigeminal ganglia and 6 mm biopsy punches of periocular skin were removed and placed in preweighed tubes containing 1 mm glass beads and 1 mL of medium. Trigeminal ganglia and periocular skin homogenates were prepared by freezing and thawing the samples, mechanically disrupting in a Mini-Beadbeater-8 (Biospec Products, Bartlesville, Oklahoma), and sonicating. Homogenates were assayed for virus by standard plaque assay, and the amount of virus was expressed as PFU per milliliter of tissue homogenate.

### 2.7. Assays of Antibody Titers

Serum was collected from mice at weekly intervals following infection and examined for HSV-specific antibody content as previously described [26]. Briefly, for enzyme linked immunosorbent assays (ELISA), serial fourfold dilutions of mouse serum were incubated for 2 hours in duplicate wells of a 96-well plate coated with purified HSV-1 glycoprotein. Biotinylated goat anti-mouse IgG was subsequently used in a colorimetric assay to determine specific IgG amounts based on comparison to a standard curve generated as previously described [26].

### 2.8. Real-Time PCR Analysis for Herpes Genome

DNA was isolated using a DNeasy tissue preparation kit (Qiagen, Valencia, CA). The number of latent genomes per trigeminal ganglion was determine by real-time PCR essentially as described [27]. Briefly, a 70 bp fragment of the thymidine kinase (tk) gene was amplified from trigeminal ganglia DNA and 10-fold dilutions of purified HSV-1 chromosome DNA. HSV-1 DNA was used to generate a standard curve to determine the number of genome copies per trigeminal ganglion models the episomal, latent genome. To control for total DNA content of each sample, the single-copy mouse adipsin gene was amplified in each sample along with dilutions of mouse genomic DNA to generate a standard curve. The values for tk copy number were normalized to the lowest value of mouse adipsin copy number to yield the normalized genome copy per ganglion, which were expressed on a log scale.

### 2.9. Flow Cytometric Analysis

Cells were isolated from corneas as previously described [18]. Briefly, corneas were excised at 18 and 23 dpi and incubated in PBS-EDTA at 37°C for 15 minutes at 37°C. Stromas were separated from overlying epithelium and digested in 84 U collagenase type 1 (Sigma-Aldrich, St. Louis, MO) per cornea for 2 hours at 37°C and then were triturated to form a single-cell suspension. Suspensions were filtered through a 40-*μ*m cell strainer cap (BD Labware, Bedford, MA) and washed and then stained. Suspensions were stained with PerCP-conjugated anti-CD45 (30-F11) and Alexa Fluor700-Gr-1 (RB6-8C5) (from BioLegend, San Diego, CA); FITC conjugated anti-CD4 (RM4-5), PE-conjugated anti-CD8*α* (53–6.7), PE-Cy7-conjugated anti-CD11c (HL3) (all BD PharMingen); eFluor450-conjugated CD11b (M1/70) (from eBiosciences, San Diego, CA). Cells were then analyzed on a flow cytometer (FACSAria with FACSDIVA data analysis software; BD Biosciences).

### 2.10. In Vitro Killing of L1210 Cells Transfected with Human Fas by Mouse Corneas

The use of whole corneas in vitro to induce Fas-mediated killing has been described [10, 14]. Mouse corneas were placed in 24-well plates with either the endothelium or epithelium facing up. These corneas were infected with the KOS strain of HSV-1 or not. To them were added L1210 cells, which express Fas (2 × 10^5^/mL were labeled with 5 *μ*Ci/mL ^3^H-thymidine at 37°C in complete DMEM for 2 hours). After washing twice they were incubated (2 × 10^4^ cells/determination) with corneas overnight in a 96-well plate overnight at 37°C. The L1210-Fas target cells were harvested onto microfiber filters and radioactivity counted using a microplate scintillation counter (TopCount, PerkinElmer Life Sciences, Boston, MA). Because fragmented DNA associated with apoptosis does not bind to the filters, the counts associated with the filters reflect nonapoptotic cell DNA only. The percentage of cells undergoing apoptosis is therefore defined as

(1) %  DNA Fragmentation=CPM L1210-Fas incubated alone−CPM L1210-Fas incubated with corneaCPM L1210-Fas incubated alone×100.

To confirm that cell killing is due to Fas-FasL interactions, we added the competitive inhibitor Fas-Fc or an inhibitor of TNF-mediated killing, TNFR1-Fc, at 10 *μ*g/mL (both from R&D Systems, Minneapolis, MN) as previously described [10, 14].

### 2.11. Statistical Analysis

All statistical analyses were performed with the aid of Sigma Stat for Windows, version 2.0 (Jandel, Corte Madera, CA). The Rank Sum test was used to compare corneal disease scores. Student's unpaired *t*-test was used to compare virus titer and antibody titer data. Fisher's exact *X* ^2^ tests were used to compare limiting dilution assay data.

## 3. Results

### 3.1. Infection of Murine Corneas Results in Increased Fas Ligand Expression

**Figure 1:**
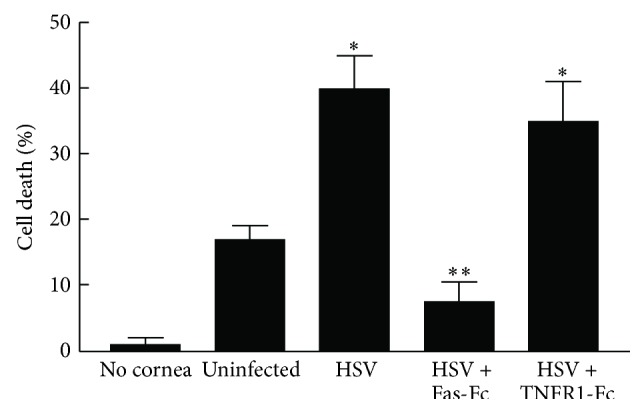
Infection with HSV-1 induces functional Fas ligand. Corneas were infected with HSV-1, KOS strain, for 24 hours at which point [^3^H]Thymidine labeled L1210-Fas cells (2 × 10^4^) were cultured in contact with the corneal epithelium in vitro for 20 h at 37°C. In some cultures the chimeric protein Fas-Fc (10 *μ*g/mL) or TNFR1-Fc (10 *μ*g/mL) was included for the entire incubation period. The amount of apoptotic cell death was determined by calculating percent of DNA fragmentation. Each value represents the mean of three replicate cultures ± SEM, and each culture condition was performed at least three times. ^*∗*^Infected corneas displayed significantly greater cell death than did uninfected corneas (*P* < 0.02). ^*∗∗*^Addition of Fas-Fc significantly reduced cell death when compared to either HSV alone (*P* < 0.01) or HSV + TNFR1-Fc (*P* < 0.02).

The first thing that we wished to determine was whether infection with HSV-1 had any effect on the expression of FasL on the cornea. Recent reports have demonstrated that FasL can be induced when cells are exposed to cytokines and stress [28, 29]. In order to do this we performed a functional assay to determine the ability of mouse corneas to kill Fas-expressing target cells. Thus we infected isolated mouse corneas with HSV-1 and then compared the ability of infected corneas to kill Fas targets versus uninfected corneas. As demonstrated in Figure 1, infected corneas were able to kill significantly more Fas-expressing target cells than uninfected corneas. We further demonstrated that this killing was due to increased FasL expression as Fas-Fc, which specifically interacts with FasL blocked killing while TNF-Fc did not (Figure 1). Since these isolated corneas do not contain significant numbers of CD45^+^ cells at the time of infection, increased FasL expression will primarily be on the resident epithelial cells. Thus it is clear that one way that the cornea attempts to limit inflammation following HSV-1 infection is by increasing FasL expression which will more efficiently control the entrance of Fas-expressing inflammatory cells.

### 3.2. Mice with Mutations in Fas or FasL Have Worse Corneal Disease

**Figure 2:**
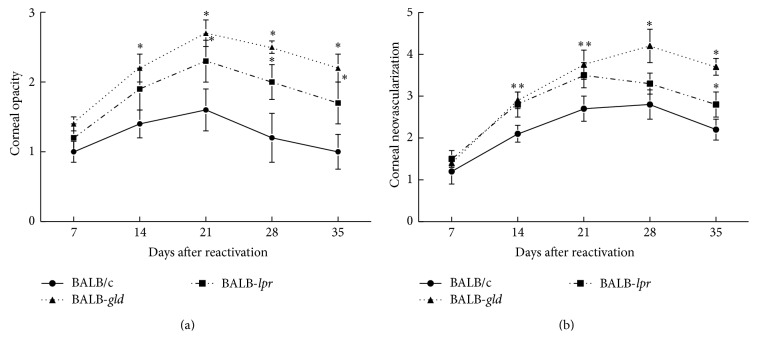
Defective expression of both FasL and to a lesser extent Fas results in increased recurrent HSK following UV-reactivation of a latent infection with HSV-1, McKrae strain. Eyes of BALB/c wild-type (*n* = 20), BALB-*lpr* (*n* = 20), and BALB-*gld* (*n* = 20) mice were infected with 10^6^ pfu of HSV-1, McKrae strain. Six weeks following infection mice were irradiated with UV-B to reactivate the latent infection. Corneal opacity (a) and corneal neovascularization (b) were measured and compared between these strains of mice. ^*∗*^Significant virus-induced corneal opacity was observed for BALB-*gld* at days 14–28 time points when compared to BALB/c controls (*P* < 0.01–0.001). BALB-*lpr* mice displayed significantly more opacity than did BALB/c controls at days 21–35 (*P* < 0.05–0.01). BALB-*gld* displayed significantly greater neovascularization at days 14–35 (*P* < 0.05–0.001) and BALB-*lpr* mice had greater neovascularization at days 14, 21, and 35 (*P* < 0.05–0.01) than did BALB/c controls.

**Figure 3:**
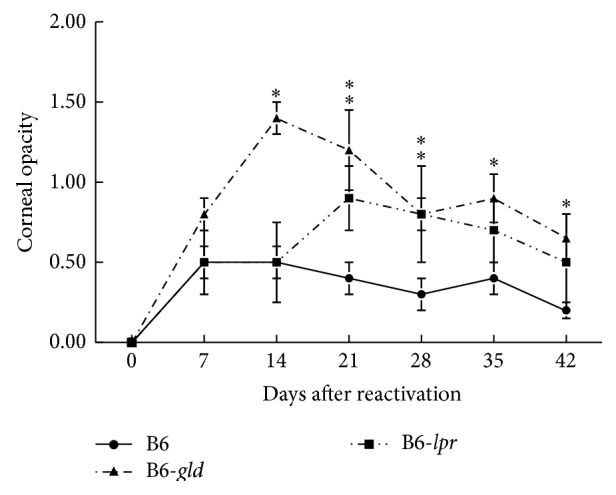
Similar patterns of disease were seen in C57BL/6 (B6) mice carrying the* gld* and* lpr* mutations following UV-B reactivation. Eyes of B6 wild-type (*n* = 20), B6-*lpr* (*n* = 15), and B6-*gld* (*n* = 15) mice were infected with 10^6^ pfu of HSV-1, McKrae strain. Six weeks following infection mice were irradiated with UV-B to reactivate the latent infection. Corneal opacity was measured and compared between these strains of mice. ^*∗*^Significant virus-induced corneal opacity was observed for B6-*gld* (*P* < 0.01–0.001) at days 14–42 when compared to B6 controls. B6-*lpr* mice displayed significantly more opacity than did B6 controls at days 21 and 28 (*P* < 0.05–0.01).

We next compared recurrent HSK between BALB-*lpr*, BALB-*gld*, and parental BALB/c mice. The mice were infected with the McKrae strain of HSV-1 and latency was established. The mice were reactivated 8 weeks following primary infection. As can be observed in Figure 2, both BALB-*lpr* and BALB-*gld* mice experienced significantly worse disease than did wild-type BALB/c mice. This was consistent with results of primary disease [18], though mice carrying the* gld* mutation tended to display consistently greater disease scores than did mice with the* lpr* mutation. A similar pattern of disease was seen in B6 mice that express mutations in Fas and FasL (Figure 3).

### 3.3. BALB/c Mice with Mutations in Fas and FasL Have Increased Mortality

**Figure 4:**
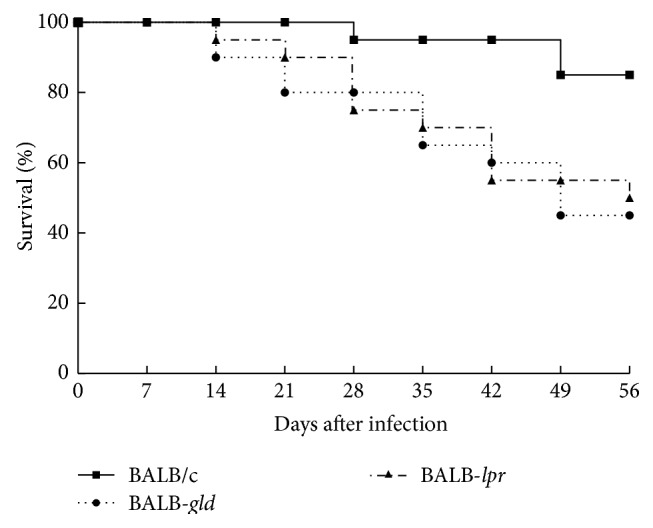
Mice expressing mutations in Fas and FasL display significantly greater mortality than wild-type BALB/c mice. A latent infection of BALB/c wild-type (*n* = 20 mice), BALB-*lpr* (*n* = 20 mice), and BALB-*gld* (*n* = 20 mice) mice was established as described and mice were observed for mortality for 10 weeks. Data displayed were compiled from two independent studies. Mortality for both BALB-*lpr* and BALB-*gld* was significantly greater than that for BALB/c wild-type mice (*P* < 0.002).

In another significant departure from our previous report, mortality was much greater in those mice carrying either the* lpr* or the* gld* mutation (Figure 4). It should be noted that the previous report compared C57BL/6 with mutations in Fas and FasL with their parental B6, but it was the same strain of HSV-1, namely, McKrae. The reason for this discrepancy is not known at this time; we suspect that BALB/c mice, which are more susceptible to both corneal disease and developing a lethal infection, are more prone to lethal infection in the absence of Fas-FasL interactions because there is greater influx of inflammatory cells into the brain (data not shown).

### 3.4. The Magnitude of the Inflammatory Infiltrate in Mice with Mutations in Fas and FasL Is Greater Than Wild-Type Mice but the Composition of the Infiltrate Is the Same

**Figure 5:**
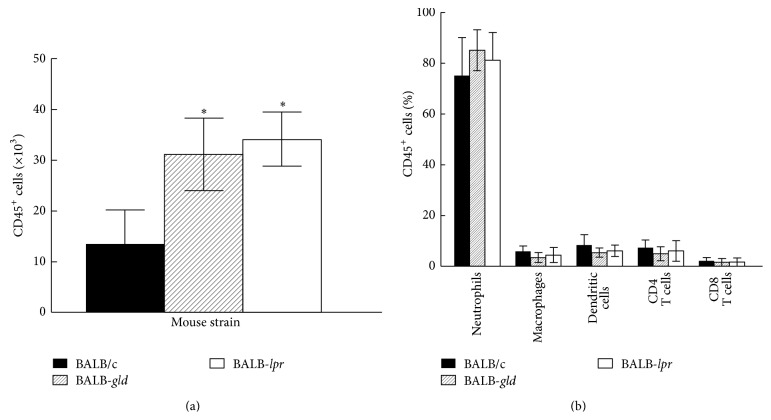
Inflammatory infiltrate in the corneas of BALB-*gld* and BALB-*lpr* mice displays significant increased CD45^+^ cells, though there are no qualitative differences in subpopulations of CD45^+^ cells. Mice were infected in one eye with HSV-1 and reactivated 8 weeks later by UV-B irradiation. The HSV-infected corneas were removed at days 17 and 23 after irradiation from mice with severe HSK disease and disaggregated into single-cell suspensions and stained with anti-CD45 (a). The CD45^+^ cells were gated and further analyzed for staining with anti-CD4, CD8*α*, Gr-1, CD11b, CD11c, and F4/80 mAb (b). Cells were analyzed by flow cytometry. Data represents 4 to 6 corneas per group. Significant differences were seen in CD45^+^ cells (a) (*P* < 0.05–0.02), but not for the percentages of CD45^+^ subsets (b) (*P* > 0.05).

We also compared the influx of inflammatory cells into the cornea of these strains of mice and as expected there were consistently more CD45^+^ cells infiltrating the corneas of* lpr* and* gld* mice than in wild-type mice following UV-induced reactivation (Figure 5(a)). We believe that is due to better control of the inflammatory infiltrate in wild-type mice that have an intact Fas-FasL interaction. However, since there are no differences in the phenotype of the inflammatory infiltrate (Figure 5(b)), there does not appear to be a differential sensitivity to Fas-FasL-mediated killing among the different types of cells that make up that infiltrate. Furthermore, those mice that display significant recurrent disease demonstrate a predominance of neutrophils regardless of the strain of mouse (Figure 5(b)). Thus, it would appear that once significant inflammation begins, the make-up of the infiltrate will be relatively the same regardless of any mutations to Fas or FasL. It should also be pointed out that serum HSV-1 specific antibody titers were indistinguishable between these groups of mice (data not shown).

### 3.5. Mice with Mutations in Fas and FasL Have Increased Reactivation and Virus at the Cornea but Do Not Show Differences in Infection of the Trigeminal Ganglia

**Figure 6:**
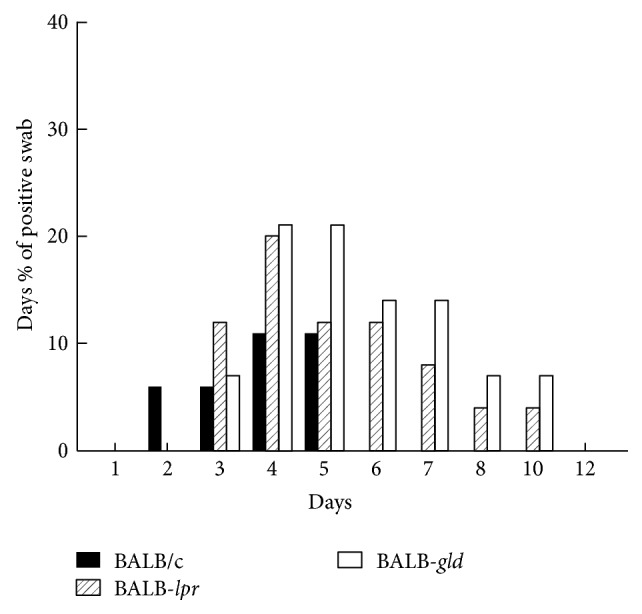
Daily percent of mice that were shedding virus following UV-B reactivation. Mice were reactivated by UV-B irradiation and swabbed daily and the presence of virus for each sample tested. Mice containing mutations in Fas (*lpr*) and FasL (*gld*) shed virus significantly longer than wild-type BALB/c mice (*P* < 0.01).

Interestingly, when these mice were compared for their ability to shed virus following UV-induced reactivation, wild-type BALB/c mice displayed reduced rates of shedding, total number of days of shedding, duration of shedding, and number of days of shedding/mouse for positive mice when compared to BALB-*lpr* and BALB-*gld* mice (Table 1). Furthermore, mice carrying the* lpr* and* gld* mutation consistently shed virus longer than did wild-type BALB/c mice (Figure 6). In contrast, the number of viral genomes in trigeminal ganglia was very similar for all strains (Table 1). Likewise the viral titers at day 3 after reactivation did not display significant differences between wild-type BALB/c mice and those carrying either the* lpr* or* gld* mutation (Table 1).

## 4. Discussion

Herpetic stromal keratitis in the human is primarily a disease that results from reactivation of HSV-1 from latently infected trigeminal ganglia neurons [1, 19, 30]. This disease is also characterized by an immunopathologic attack on the cornea following such reactivations [19, 30]. The good news is that most individuals harboring a latent infection of the trigeminal ganglia probably never exhibit overt clinical disease. Thus it is clear that should virus be reactivated in these clinically silent individuals their immune response does not result in a damaging inflammatory response to the cornea. This is likely the result of several factors, some of which are driven by the immune response towards HSV-1 that develops in the infected individual and some due to intrinsic factors within the cornea that strive to limit strong inflammatory responses. One of the prime mechanisms the eye uses to protect itself from T cell-mediated immunopathologic response is the presence of FasL which induces apoptosis in Fas^+^ lymphoid cells [8, 9, 11, 12]. Consequently, those factors that either directly or indirectly lead to changes in FasL expression would greatly affect the cornea's ability to restrict inflammatory infiltrates. It is known that some factors, such as inhibitors of matrix metalloproteases, stabilize FasL expression which results in increased surface expression of FasL and a concomitant increase in the ability of the cornea or the choroid to control inflammatory [31] and angiogenic invasion [32]. Likewise the production of IL-18 has also been recently shown to induce FasL expression [29]. We report here that ex vivo infection of corneas with HSV-1 also increases the functional expression of FasL. Thus one might speculate that one way that the cornea attempts to control damaging inflammatory invasion is by increasing FasL expression following exposure to infectious agents.

We and others have reported that lack of functional Fas-FasL-mediated apoptotic ability in the eye most often leads to increased inflammatory responses [8, 9, 12], increased corneal allograft rejection [12, 33], increased acute HSK [18], increased neovascularization [10, 13, 14], and the inability to develop systemic tolerance following injection of antigen into the anterior chamber [8]. Thus it is not surprising that mice that are defective in Fas-FasL display increased recurrent HSK.

This observation is partially consistent with what was observed in these mice during acute HSK [18]. Namely, they experience significantly increased disease when compared to wild-type mice. In spite of the general similarity in results there were some potentially important differences. First, recurrent disease was more pronounced in* gld* mice than that observed in* lpr* mice. This difference, while not statistically significant, was consistent for mice on both the BALB/c and the B6 backgrounds. The reason for this difference has not been determined and will be the subject of future studies. None the less it might relate to the fact that* gld* mice do not control neovascularization as well as* lpr* mice do [14]. It was determined that this was a consequence of the leakiness of the* lpr* mutation in terms of vascular endothelium's expression of Fas [14]. Consequently, while both* lpr* and* gld* mice would demonstrate a similar lack of control of inflammatory cell infiltrate,* gld* mice would have more neovascularization which could result in overall greater disease. In addition, since infection with HSV-1 increases the expression of FasL on the cornea, one might expect the* gld* phenotype to have a more pronounced effect on controlling any population of Fas-expressing cells to enter the infected cornea.

In addition to the minor differences in corneal disease between* lpr* and* gld* mice was the remarkable difference in mortality seen between BALB/c and both BALB-*lpr* and BALB-*gld* mice when they were infected with the neurovirulent McKrae strain of HSV-1. What is even more surprising was that this difference in mortality occurs despite the fact that all strains of mice were provided with neutralizing antibody. We had previously reported that* lpr* and* gld* mice on the B6 background that were acutely infected with the McKrae strain of HSV-1 were more resistant than wild-type B6 mice [18]. This intriguing strain-associated difference could be a clue to how different genetic backgrounds can greatly alter the ability of the HSV-1 for traffic within the infected host. We had believed that the relative resistance seen in B6-*lpr* and B6-*gld* mice was due to the lack of apoptotic cell death that might be inducing tolerance towards HSV-1 as has been reported for other types of infections [34, 35]. This does not appear to be the case with Fas/FasL mutations on the BALB/c background. Therefore, it is entirely possible that the increased inflammation seen in the cornea may also occur wherever the virus spreads and thus play a role in compromising the blood-brain barrier allowing increased viral penetration of the brain followed by entrance of inflammatory cells leading to death by encephalitis. At present we do not have direct data indicating that this is the case, but we are currently planning studies to better understand the increased mortality associated with BALB/c mice expressing mutations in Fas or FasL.

In addition to these anti-inflammatory responses that are specific to the eye, it is also well established that host T cells eliminate viral infected cells either by the perforin-granzyme pathway [36] or via apoptosis mediated by the interaction of FasL on effector cells with Fas expressed by virally infected cells [37, 38]. Thus it is also possible that mice defective in killing via Fas-FasL would display reduced ability to kill virally infected targets. This could be one of the reasons why virus persists in the corneas of both* gld* and* lpr* mice. It might also provide another explanation for why* gld* and* lpr* mice have increased mortality when infected with the McKrae strain of HSV-1. That said, lack of a functional Fas-FasL interaction does not prevent these mice from clearing infectious virus from the cornea as evidenced by the fact that infectious virus is not detectible in the corneas of reactivated mice after 10 days following reactivation.

It should also be pointed out that characterization of the inflammatory infiltrate in mice suffering from significant corneal disease illustrates two concepts. The first is that, overall, the corneas of wild-type BALB/c mice have quantitatively fewer CD45^+^ cells than do those mice with mutations in Fas or FasL. Secondly, when mice with significant disease are analyzed qualitatively there are no significant qualitative differences between wild-type and mutant mice (Figure 5). All mice with significant disease, regardless of their genotype, have infiltrates that consist of large numbers of neutrophils and much lower numbers of T cells, macrophages, and dendritic cells. Thus, once again, it appears that corneal disease is best associated with the presence of large numbers of neutrophils that infiltrate the cornea [4, 18].

Previous work from this and other investigators have shown that development of an antibody response against HSV-1 can protect mice from the development of severe HSK [20, 39, 40]. As a consequence we tested antibody responses in BALB/c, BALB-*lpr,* and BALB-*gld* mice to determine if* lpr* or* gld* mice had impaired anti-HSV-1 responses. However, no differences were observed between these strains (data not shown), indicating that the ability to develop an anti-HSV-1 antibody response was not involved.

Taken together, these studies document that mice with impaired Fas-FasL interactions develop significantly increased HSK following UV-B induced reactivation. This response is slightly more pronounced in mice with mutations in FasL than in Fas. The mechanism responsible for increased disease is likely due to increased inflammation of the cornea not by the qualitative nature of that inflammation. This Increased inflammation is likely driven by two factors: one is the reduced control of infiltrating inflammatory cells and second a prolonged presence of infectious virus in the cornea.

## Figures and Tables

**Table 1:** HSV-1 shedding following UV-B reactivation.

	Normal BALB/c	BALB-*gld *	BALB-*lpr *
EYES			
% of positive swabs^@^	11%	17%	23%
Total shedding days^#^	7	22	41
Days of shedding/mouse^†^	1.2 ± 0.3	2.8 ± 0.25	2.9 + 0.6
Reactivation rate^‡^	33% (6/18 total)	57% (8/14 total)	54% (14/26 total)
Final day of shedding^||^	Day 5	Day 10	Day 10
Trigeminal ganglia			
Genome copies × 10^2^ (*n*)^*∗∗*^	6.5 ± 0.3 (10)	10.4 ± 0.4 (5)	11.1 + 0.3 (5)
Titer day 3 after react. (*n*)^††^	845 ± 217 (4)	1186 ± 159 (4)	1075 + 245 (4)

^@^The percent of positive swabs is the percentage of virus-positive eye swabs (140 to 286 eye swabs per group) over the 10-day period following UV-B irradiation. (*P* < 0.01 for both *gld* and *lpr* mice.)

^#^Total shedding days: number of days of positive swab. (*P* < 0.01 for both *gld* and *lpr* mice.)

^†^Days of shedding/mouse are the number of days that a positive mouse shed virus. (*P* < 0.005 for both *gld* and *lpr* mice.)

^‡^Percentage of reactivation rate is the percentage of mice that were reactivated. (*P* < 0.02 for both *gld* and *lpr* mice.)

^||^Final day of shedding was the last day that a mouse was positive for a particular group.

^*∗∗*^Mean number of genome copies from real-time PCR analysis. Statistical analysis did not indicate significant differences (*P* > 0.05).

^††^Titer of virus at day 3 after reactivation.

## References

[1] Pepose J. S., Leib D. A., Stuart P. M., Easty E. L. (1996). Herpes simplex virus diseases: anterior segment of the eye. *Ocular Infection and Immunity*.

[2] Thomas J., Gangappa S., Kanangat S., Rouse B. T. (1997). On the essential involvement of neutrophils in the immunopathologic disease: herpetic stromal keratitis. *Journal of Immunology*.

[3] Maertzdorf J., Verjans G. M. G. M., Remeijer L., van der Kooi A., Osterhaus A. D. M. E. (2003). Restricted T cell receptor *β*-chain variable region protein use by cornea-derived CD4^+^ and CD8^+^ herpes simplex vires-specific T cells in patients with herpetic stromal keratitis. *Journal of Infectious Diseases*.

[4] Divito S. J., Hendricks R. L. (2008). Activated inflammatory infiltrate in HSV-1-infected corneas without herpes stromal keratitis. *Investigative Ophthalmology & Visual Science*.

[5] Denniston A. K., Kottoor S. H., Khan I. (2011). Endogenous cortisol and TGF-*β* in human aqueous humor contribute to ocular immune privilege by regulating dendritic cell function. *Journal of Immunology*.

[6] Cursiefen C. (2007). Immune privilege and angiogenic privilege of the cornea. *Chemical Immunology and Allergy*.

[7] Koevary S. B. (2000). Ocular immune privilege: a review. *Clinical Eye and Vision Care*.

[8] Griffith T. S., Brunner T., Fletcher S. M., Green D. R., Ferguson T. A. (1995). Fas ligand-induced apoptosis as a mechanism of immune privilege. *Science*.

[9] Griffith T. S., Yu X., Herndon J. M., Green D. R., Ferguson T. A. (1996). CD95-induced apoptosis of lymphocytes in an immune privileged site induces immunological tolerance. *Immunity*.

[10] Kaplan H. J., Leibole M. A., Tezel T., Ferguson T. A. (1999). Fas ligand (CD95 ligand) controls angiogenesis beneath the retina. *Nature Medicine*.

[11] Osawa H., Maruyama K., Streilein J. W. (2004). CD95 ligand expression on corneal epithelium and endothelium influences the fates of orthotopic and heterotopic corneal allografts in mice. *Investigative Ophthalmology and Visual Science*.

[12] Stuart P. M., Griffith T. S., Usui N., Pepose J., Yu X., Ferguson T. A. (1997). CD95 ligand (FasL)-induced apoptosis is necessary for corneal allograft survival. *Journal of Clinical Investigation*.

[13] Volpert O. V., Zaichuk T., Zhou W. (2002). Inducer-stimulated Fas targets activated endothelium for destruction by anti-angiogenic thrombospondin-1 and pigment epithelium-derived factor. *Nature Medicine*.

[14] Stuart P. M., Pan F., Plambeck S., Ferguson T. A. (2003). Fas/Fas ligand interactions regulate neovascularization in the cornea. *Investigative Ophthalmology and Visual Science*.

[15] Richardson B. C., Lalwani N. D., Johnson K. J., Marks R. M. (1994). Fas ligation triggers apoptosis in macrophages but not endothelial cells. *European Journal of Immunology*.

[16] Laurence J., Mitra D., Steiner M., Staiano-Coico L., Jaffe E. (1996). Plasma from patients with idiopathic and human immunodeficiency virus-associated thrombotic thrombocytopenic purpura induces apoptosis in microvascular endothelial cells. *Blood*.

[17] Suhara T., Fukuo K., Sugimoto T. (1998). Hydrogen peroxide induces up-regulation of Fas in human endothelial cells. *Journal of Immunology*.

[18] Morris J. E., Zobell S., Yin X.-T. (2012). Mice with mutations in fas and fas ligand demonstrate increased herpetic stromal keratitis following corneal infection with HSV-1. *The Journal of Immunology*.

[19] Stuart P. M., Keadle T. L. (2012). Recurrent herpetic stromal keratitis in mice: a model for studying human HSK. *Clinical and Developmental Immunology*.

[20] Keadle T. L., Morrison L. A., Morris J. L., Pepose J. S., Stuart P. M. (2002). Therapeutic immunization with a virion host shutoff-defective, replication-incompetent herpes simplex virus type 1 strain limits recurrent herpetic ocular infection. *Journal of Virology*.

[21] Stuart P. M., Yin X. T., Plambeck S., Pan F., Ferguson T. A. (2005). The role of Fas ligand as an effector molecule in corneal graft rejection. *European Journal of Immunology*.

[22] Herndon J. M., Stuart P. M., Ferguson T. A. (2005). Peripheral deletion of antigen-specific T cells leads to long-term tolerance mediated by CD8^+^ cytotoxic cells. *The Journal of Immunology*.

[23] Stuart P. M., Summers B., Morris J. E., Morrison L. A., Leib D. A. (2004). CD8^+^ T cells control corneal disease following ocular infection with herpes simplex virus type 1. *Journal of General Virology*.

[24] Keadle T. L., Laycock K. A., Miller J. K. (1997). Efficacy of a recombinant glycoprotein D subunit vaccine on the development of primary and recurrent ocular infection with herpes simplex virus type 1 in mice. *Journal of Infectious Diseases*.

[25] Smith T. J., Ackland-Berglund C. E., Leib D. A. (2000). Herpes simplex virus virion host shutoff (*vhs*) activity alters periocular disease in mice. *Journal of Virology*.

[26] Geiss B. J., Smith T. J., Leib D. A., Morrison L. A. (2000). Disruption of virion host shutoff activity improves the immunogenicity and protective capacity of a replication-incompetent herpes simplex virus type 1 vaccine strain. *Journal of Virology*.

[27] Strand S. S., Leib D. A. (2004). Role of the VP16-binding domain of vhs in viral growth, host shutoff activity, and pathogenesis. *Journal of Virology*.

[28] Lin J. C., Peng Y. J., Wang S. Y., Young T. H., Salter D. M., Lee H. S. (2015). Role of the sympathetic nervous system in carbon tetrachloride-induced and systemic inflammation. *PLoS ONE*.

[29] Yano T., Nozaki Y., Kinoshita K. (2015). The pathological role of IL-18R*α* in renal ischemia/reperfusion injury. *Laboratory Investigation*.

[30] Liesegang T. J. (2001). Herpes simplex virus epidemiology and ocular importance. *Cornea*.

[31] Stuart P. M., Pan F., Yin X. T., Haskova Z., Plambeck S., Ferguson T. A. (2004). Effect of metalloprotease inhibitors on corneal allograft survival. *Investigative Ophthalmology and Visual Science*.

[32] Roychoudhury J., Herndon J. M., Yin J., Apte R. S., Ferguson T. A. (2010). Targeting immune privilege to prevent pathogenic neovascularization. *Investigative Ophthalmology and Visual Science*.

[33] Yamagami S., Kawashima H., Tsuru T. (1997). Role of Fas-Fas ligand interactions in the immunorejection of allogeneic mouse corneal transplants. *Transplantation*.

[34] Summerfield A., McNeilly F., Walker I., Allan G., Knoetig S. M., McCullough K. C. (2001). Depletion of CD4+ and CD8^high+^ T-cells before the onset of viraemia during classical swine fever. *Veterinary Immunology and Immunopathology*.

[35] Chung C.-S., Song G. Y., Lomas J., Simms H. H., Chaudry I. H., Ayala A. (2003). Inhibition of Fas/Fas ligand signaling improves septic survival: differential effects on macrophage apoptotic and functional capacity. *Journal of Leukocyte Biology*.

[36] Ishikawa T., Yamada H., Oyamada A., Goshima F., Nishiyama Y., Yoshikai Y. (2009). Protective role of Fas-FasL signaling in lethal infection with herpes simplex virus type 2 in mice. *Journal of Virology*.

[37] Rouvier E., Luciani M. F., Golstein P. (1993). Fas involvement in Ca^2+^-independent T cell-mediated cytotoxicity. *The Journal of Experimental Medicine*.

[38] Dobbs M. E., Strasser J. E., Chu C.-F., Chalk C., Milligan G. N. (2005). Clearance of herpes simplex virus type 2 by CD8^+^ T cells requires gamma interferon and either perforin- or Fas-mediated cytolytic mechanisms. *Journal of Virology*.

[39] Deshpande S. P., Zheng M., Daheshia M., Rouse B. T. (2000). Pathogenesis of herpes simplex virus-induced ocular immunoinflammatory lesions in B-cell-deficient mice. *Journal of Virology*.

[40] Hu K., Dou J., Yu F. (2011). An ocular mucosal administration of nanoparticles containing DNA vaccine pRSC-gD-IL-21 confers protection against mucosal challenge with herpes simplex virus type 1 in mice. *Vaccine*.

